# Differential chromatin proteomics of the MMS-induced DNA damage response in yeast

**DOI:** 10.1186/1477-5956-9-62

**Published:** 2011-10-04

**Authors:** Dong Ryoung Kim, Rohan D Gidvani, Brian P Ingalls, Bernard P Duncker, Brendan J McConkey

**Affiliations:** 1 Department of Biology, University of Waterloo, 200 University Avenue, Waterloo, ON, Canada; 2 Department of Applied Mathematics, University of Waterloo, 200 University Avenue, Waterloo, ON, Canada

**Keywords:** chromatin, fractionation, DIGE, differential, proteomics, MMS, DNA damage

## Abstract

**Background:**

Protein enrichment by sub-cellular fractionation was combined with differential-in-gel-electrophoresis (DIGE) to address the detection of the low abundance chromatin proteins in the budding yeast proteome. Comparisons of whole-cell extracts and chromatin fractions were used to provide a measure of the degree of chromatin association for individual proteins, which could be compared across sample treatments. The method was applied to analyze the effect of the DNA damaging agent methyl methanesulfonate (MMS) on levels of chromatin-associated proteins.

**Results:**

Up-regulation of several previously characterized DNA damage checkpoint-regulated proteins, such as Rnr4, Rpa1 and Rpa2, was observed. In addition, several novel DNA damage responsive proteins were identified and assessed for genotoxic sensitivity using either DAmP (decreased abundance by mRNA perturbation) or knockout strains, including Acf2, Arp3, Bmh1, Hsp31, Lsp1, Pst2, Rnr4, Rpa1, Rpa2, Ste4, Ycp4 and Yrb1. A strain in which the expression of the Ran-GTPase binding protein Yrb1 was reduced was found to be hypersensitive to genotoxic stress.

**Conclusion:**

The described method was effective at unveiling chromatin-associated proteins that are less likely to be detected in the absence of fractionation. Several novel proteins with altered chromatin abundance were identified including Yrb1, pointing to a role for this nuclear import associated protein in DNA damage response.

## Background

Within many proteomic studies, protein abundance and complexity can affect practical detection sensitivity, even with advances in differential in-gel electrophoresis (DIGE) [1] and MS-based approaches [2]. For example, certain functional classes of proteins such as transcription factors and cell cycle proteins are present at low abundance in whole cell extracts compared to other structural and metabolic proteins [3]. In response to the issues of low abundance and dynamic range limitations of quantitative proteomics methods (e.g. LC-MS or DIGE), one strategy is to minimize sample complexity through enrichment approaches, such as affinity capture of protein complexes (e.g. tandem affinity purification) [4], selection of phosphopeptides [5], and sub-cellular fractionation [6-8]. Although targeted affinity-based methods can lead to high levels of enrichment, they have a high probability of excluding relevant proteins. An attractive alternative approach is a sub-cellular fractionation, where overall protein complexity and stoichiometry can be largely retained during the fractionation. Based on this rationale, cellular organelles have been subjected to proteomic analysis, including mitochondria and chloroplasts [6-8], demonstrating that the combination of sub-cellular fractionation and proteomics techniques provides a practical means for the analysis of low-abundance proteins localized in discrete regions of the cell.

Though it is not a separate organelle per se, chromatin is physically organized in the cell and, due to the importance of chromatin in molecular analyses of DNA replication and epigenetics, procedures to separate chromatin from other cellular components have become well established in budding yeast [9-11]. By using fractionated chromatin samples, MS-based approaches have been employed to identify a wide range of chromatin-associated proteins, including those from developing *Xenopus *embryos [12] and *C. elegans *sperm [13]. As demonstrated in such studies based on chromatography and/or mass spectrometry-based analysis of digested peptides, initial fractionation coupled with downstream proteomics methods is extremely valuable for addressing the relatively low abundance of many chromatin-associated proteins, especially in the context of large-scale protein identification. However, it can still be challenging to address differential expression using fractionated chromatin, as technical variability during its preparation can interfere with multiplex sampling and stringent statistical evaluation is needed to minimize false discovery rates. In addressing this aspect, gel-based proteomics is a promising approach to accommodate multiplex experimentation effectively while minimizing systemic experimental variation. In addition, the DIGE method is extremely useful for identifying various protein forms resulting from posttranslational modifications such as phosphorylation [14] and evaluating their relative abundance.

Chromatin-associated proteins mediate a multitude of biological processes such as DNA replication, repair, and transcription [15-17], through complex regulatory mechanisms. The structure of chromatin changes as a function of the cell cycle, adopting a more condensed conformation during mitotic phase relative to interphase, when DNA is duplicated. When chromatin integrity is compromised as a result of exposure to genotoxic agents, the cellular repair machinery is recruited to sites of DNA damage [18,19]. The appropriate regulation of each process requires a multitude of mechanisms such as histone modification [20], chromatin remodeling [21], and formation of diverse protein complexes. In studies of biological mechanisms, the qualitative and quantitative analyses of interactions and/or binding with chromatin are crucial in order to investigate protein function, signaling pathways, and modular networks [22]. Therefore, global proteomic profiling of chromatin provides an effective means to gain valuable information about these central biological processes [22], and has widespread applications such as acceleration of pharmaceutical development [20].

In this study, we have conducted an analysis of differential protein expression using 2D-DIGE in combination with chromatin fractionation of budding yeast. We first assessed the effectiveness of our approach in isolating and detecting chromatin-associated proteins using DIGE. The combination of DIGE with fractionation allows both identification of differential abundance due to an applied treatment, and additionally provides a means to estimate changes in protein localization, or in this case, chromatin affinity. The potential utility of this novel approach was then confirmed by applying the method to screen for differentially expressed proteins following treatment with the DNA damaging methyl methanesulfonate (MMS), resulting in the detection of both known and novel DNA damage response proteins.

## Results and discussion

### Initial DIGE based identification of chromatin fraction proteins

Yeast protein extracts from whole cells and from chromatin enrichment were compared using DIGE. Candidate proteins were selected for identification on the basis of chromatin enrichment factor (EF), defined for a given spot as the average ratio of spot volume in the chromatin fraction vs. the whole cell extract in DIGE images [Additional File 1, Figure S1]. Enrichment factors were calculated for paired chromatin and WCE samples in the four DIGE gels using the BVA analysis module within the DeCyder™ software package. *P*-values were also calculated for protein spots, but as two different sample types are being compared these provide only a relative measure of variability and enrichment. As the initial fractionation procedure retained 2.1% of the total cellular protein on average, the theoretical upper limit of the enrichment factor is approximately 50 fold.

**Figure 1:**
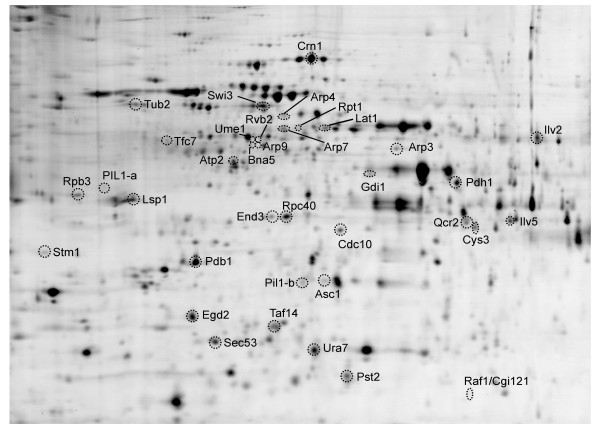
**2D-protein spot map of the yeast chromatin fraction**. Representative proteins enriched in the chromatin fraction were identified by mass spectrometry and are marked with corresponding protein names. Protein identification data are summarized in Additional File 1, Table S1 with respective chromatin enrichment factors and *p*-values.

To verify that the fractionation was successful at targeting chromatin-associated proteins, a subset of enriched protein spots was analyzed by mass spectrometry. A Coomassie-stained gel was prepared from a chromatin-enriched yeast fraction for protein identification (Figure 1). Spots with an experimental enrichment factor greater than 1.4 fold were selected for MS analysis, and 33 of these were identified (Table 1, Additional File 1, Tables S1). Based on annotations from the Saccharomyces Genome Database http://www.yeastgenome.org, the organelle database http://organelledb.lsi.umich.edu, and literature sources, the majority have been previously identified as localizing to the nucleus and include many functionally important chromatin proteins. Estimated protein copy number per cell [3] is shown in Table 1 for known chromatin-associated proteins identified in the chromatin fraction [Additional File 1, Figure S1]. Overall, the fractionation procedure was effective at enriching low-abundance chromatin associated proteins. Interestingly, a number of the identified proteins had very low expected cellular levels (e.g. 1070 copies/cell for Arp4 and 1360 for Arp7) based on previous GFP fusion experiments [3].

**Table 1:** Proteins identified within the chromatin enriched fraction.

Protein name	Enrichment factor	**Estimated copies/cell**^**a**^	**Cellular Localization**^**b**^	**Description**^**c**^
Arp3	+1.74	6650	Cytoskeleton, Nucleus [54]	Actin-related protein 3, actin filament organization

Arp4	+4.20	1070	Nucleus	Actin-related protein 4, chromatin remodeling

Arp7	+2.53	1360	Nucleus	Actin-related protein 7, chromatin remodeling

Arp9	+3.35	1790	Nucleus^d^	Actin-related protein 9, chromatin remodeling

Asc1	+2.88	333000	Cytoplasm	G protein beta subunit, Small subunit ribosomal protein

Atp2	+1.43	164000	Mitochondrion	F1-ATPase beta chain, mitochondrial ATP synthesis

Cdc10	+3.09	14100	Septin ring, cytosketelon, nucleus^d^	Septin ring protein, cell division

Cgi121	+3.37	N.D.	Nucleus [27]	Component of KEOPS, telomere uncapping and elongation

Crn1	+4.06	2900	Contractile ring, Cytoskeleton^d^	Coronin, actin filament organization

Cys3	+1.65	38300	Cytoplasm	Gamma-cystathionase, Cysteine biosynthesis

Egd2	+1.55	38000	Cytoplasm, Nucleus [55]	Component of NAC, ribosome associated

End3	+10.4	2600	Cytoskeleton	EH domain protein, actin cytoskeletal organization

Gdi1	+1.58	7280	Cytoplasm	GDP dissociation inhibitor, vesicle mediated transport

Ilv2	+13.4	31900	Mitochondrion	Acetolactate synthase, amino acid synthesis

Ilv5	+1.95	883000	Nucleus, Mitochondrion^d^	Acetohydroxy-acid isomerase, amino acid synthesis

Lat1	+2.42	5440	Mitochondrion	Dihydrolipoamide acetyl-transferase, pyruvate metabolism

Lsp1	+5.54	104000	Cytoplasm (punctate composite)	Component of eisosome, endocytosis

Pdb1	+3.93	9970	Mitochrondrion, Nucleoid^d^	Pyruvate dehydrogenase, pyruvate metabolism

Pil1	+1.65, Pil1(a) +2.31, Pil1(b)	115000	Cytoplasm (punctate composite)	Component of eisosome, endocytosis

Pst2	+3.37	2330	Mitochondrion, Nucleus [56]	Flavodoxin-like protein

Qcr2	+1.73	35700	Mitochondrion	Ubiquinol cytochrome C reductase, respiration

Raf1	+3.37	N.D.	Nucleus [57]	FLP1recombinase activating factor, plasmid maintenance

Rpb3	+3.38	10000	Nucleus	DNA directed RNA polymerase II

Rpc40	+3.98	13000	Nucleus	Component of RNA polymerases I

Rpt1	+1.92	105	Nucleus	ATPase subunit of proteosome

Rvb2	+4.03	3030	Nucleus^d^	Transcription, chromatin remodeling

Stm1	+2.21	46800	Cytoplasm, Nucleus^d^	TOR signaling, telomere structure

Swi3	+6.85	3150	Nucleus	Chromatin remodeling complex, SWI/SNF

Taf14	+4.32	3120	Nucleus	Subunit of TFIID, TFIIF, INO80, SWI/SNF, NUA3 complexes, chromatin remodeling

Tfc7	+3.89	2660	Cytoplasm, Nucleus	RNA polymerase IIIc

Tub2	+3.73	N.D.	Nucleus, Cytoskeleton^d^	Tublin 2, microtubule component

Ume1	+2.22	3040	Cytoplasm, Nucleus	Negative regulator of meosis, binding to histone deacetylase RPD3.

Ura7	+3.73	57600	Cytoplasm	CTP synthase, phospholipid biosynthesis

DIGE was used to compare the chromatin fraction vs. whole cell yeast extract, and protein spots with an enrichment factor greater than +1.40 were selected for identification.

a - Protein copy numbers per cell are from Ghaemmaghami et al. (2003) [3], obtained via the Saccharomyces Genome Database http://www.yeastgenome.org.

b - unless otherwise noted, localizations were obtained from Huh et al (2003) [58] via the Saccharomyces Genome Database.

c- functional descriptions were obtained from the Saccharomyces Genome Database.

d- localization was obtained from the organelle database http://organelledb.lsi.umich.edu.

Among the identified proteins, some belong to well-known complexes involved in chromatin remodeling, such as SWI/SNF and INO80 [23]. These include Swi3, Taf14, Arp4, Arp7, Arp9, and Rvb2. Members of RNA polymerase complexes [24,25] were also identified, including the proteins Rpc40, Rpb3, and Tfc7. In addition, some proteins important for telomere capping and remodeling were found, such as Stm1 [26] and Cgi121 [27]. In many cases, proteins were identified along with other factors they normally interact with, implying good retention and co-enrichment of complex subunits. This result strongly suggests that chromatin fractionation was effective at enriching for functional chromatin proteins.

### Changes in chromatin fraction due to MMS treatment

To further investigate differential profiling of chromatin-associated proteins, we examined the response to MMS-induced changes in budding yeast. A well-studied genotoxic agent, MMS alkylates DNA and results in activation of the DNA damage checkpoint, initially with detection of DNA damage, followed by a signaling cascade which results in the phosphorylation of protein targets involved in cell cycle control, DNA replication and repair [18,19]. Comparison of chromatin fractions from MMS treated and control samples should indicate proteins that are differentially regulated and/or have a greater degree of chromatin association in response to MMS.

Four independent replicates of cultures were made for untreated samples and samples treated with 0.03% MMS, and chromatin enrichment was conducted as before. Differential protein abundance in the chromatin fraction was compared between MMS treated and control samples using DIGE [Additional File 1 Figure S2]. Additionally, whole-cell extracts and chromatin fractions were compared to calculate protein enrichment factors in the presence of MMS. The statistical power of detecting changes in abundance was also estimated, and at a statistical power of 0.8 (β = 0.2) with α = 0.05, the four DIGE gels can theoretically be used to identify a change of 1.43 fold in spot abundance with a success rate of 80%. The normalized standard deviation of protein spots present on all gels was 0.216 for untreated samples and 0.220 for the MMS treated samples. Differential factor (DF) values for MMS treatment were determined through quantification using the DeCyder™ v.6.0 software as described in Experimental Procedures, with DF calculated from the ratio of the protein in the MMS treated sample vs. the control sample. Here, DF includes contributions from both expression and changes in localization; for example, if DF increases but the EF ratios for the MMS+ treated and control samples are similar, the change is largely due to expression. It is also possible that DF to be positive and the EF ratio to decrease, indicating an increased protein expression and increased amount in the chromatin fraction, but a larger increase in non-chromatin associated protein. A total of 1763 spots were matched across the four replicates in the differential MMS experiment, of which 455 showed significant changes (increased or decreased) at *p *< 0.05 with FDR correction. Comparing the calculated EF values from chromatin enrichment for these 455 spots, 217 were both differentially regulated and enriched in chromatin fractions.

### Identification of MMS responsive proteins

**Figure 2:**
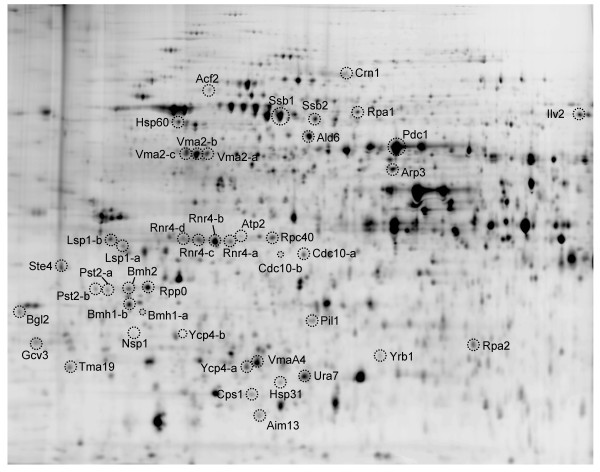
**2D-protein spot map showing differentially expressed proteins identified in the MMS treated yeast chromatin fraction**. Proteins with statistically significant changes abundance on MMS treatment were identified by mass spectrometry and are marked with corresponding protein names. Protein identification data are summarized in Additional File 1, Table S2.

Protein spots from the MMS DIGE experiment were prioritized for identification according to the degree of chromatin enrichment (EF) and changes in observed abundance ('differential factor', DF). Spots that showed both positive EF and DF values (among the 217 described above) were of particular interest, as they indicated both chromatin-association and induction by MMS treatment, respectively. A preparative Coomassie-stained gel was made using chromatin fractions of the MMS treated samples (Figure 2), and protein spots were excised for identification by mass spectrometry. Identifications were made for 23 DF+ proteins and 12 DF- proteins (Table 2, Additional File 1, Table S2). A subgroup of identified proteins corresponds to known checkpoint-regulated proteins, including Rnr4, Rpa1, and Rpa2. Rpa1 and Rpa2 are subunits of the hetero-trimeric replication factor A complex, which plays an integral role in DNA replication and checkpoint responses [19,28-30]. Among the spots with negative enrichment factors, Rnr4 isoforms exhibited some of the largest responses to MMS treatment as reflected by DF values (Table 2). The RNR complex controls the nucleotide pool for DNA synthesis and is a downstream target of the Rad53 checkpoint kinase [31,32].

**Table 2:** Statistical data for MMS-induced differentially expressed proteins in chromatin fraction.

Protein name	DF	p-value (DF)	EF (+MMS)	p-value (EF, +MMS)	EF (-MMS)	p-value (EF, -MMS)
Acf2	+1.48	0.020	+1.57	0.011	-1.30	0.048

Aim13	+1.70	0.0053	+3.30	0.034	+2.27	0.00097

Arp3	+1.35	0.024	N/A	N/A	+1.52	0.063

Atp2	+1.52	0.0093	+1.50	0.093	+5.74	0.000041

Bmh1 (a)	+1.92	0.0014	+3.57	0.0039	-1.03	0.070

Cdc10 (a)	+1.61	0.0034	+7.28	0.0099	+6.33	0.000015

Cdc10 (b)	+1.38	0.044	+1.50	0.18	+2.43	0.0015

Cps1	+1.61	0.010	+2.16	0.0036	-2.57	0.00018

Crn1	+1.78	0.0034	+3.85	0.0024	+2.36	0.0012

Gcv3	+1.75	0.019	+1.07	0.52	-1.90	0.00087

Ilv2	+2.15	0.013	+1.50	0.39	-1.15	0.086

Lsp1 (a)	+1.51	0.0023	+3.17	0.0011	+1.79	0.022

Lsp1 (b)	+1.84	0.0046	+3.69	0.00072	-1.71	0.0078

Nsp1	+1.58	0.0050	+3.07	0.014	+1.65	0.017

Pil1	+2.05	0.0013	+9.60	0.0068	+2.65	0.036

Pst2 (a)	+1.50	0.0023	+3.99	0.0074	+1.55	0.0019

Pst2 (b)	+3.83	0.0013	+4.50	0.0074	-1.02	0.14

Rpa1	+3.58	0.00070	+4.16	0.010	-1.33	N/A

Rpa2	+1.47	0.036	+2.50	0.015	-1.12	0.089

Ste4	+1.61	0.023	+2.32	0.000092	-1.00	0.16

Vma2 (a)	+1.92	0.0052	+1.59	0.0063	-1.34	0.00019

Vma2 (b)	+1.48	0.018	+1.64	0.0041	-1.58	0.00018

Vma2 (c)	+1.53	0.0097	+1.65	0.021	+1.10	0.050

Vma4	+1.85	0.010	+1.58	0.0048	-1.08	0.070

Ycp4 (a)	+2.01	0.00070	+5.40	0.00073	+1.76	0.00050

Ycp4 (b)	+1.69	0.037	+5.31	0.00063	+1.18	0.13

Yrb1	+2.05	0.025	+5.07	0.0011	+1.77	0.0040

Hsp31	+1.63	0.0063	-1.64	0.0051	-1.84	0.000062

Rnr4 (a)	+1.91	0.0014	-1.24	0.25	-1.47	0.013

Rnr4 (b)	+3.90	0.000057	-2.08	0.012	-3.11	0.00011

Rnr4 (c)	+3.89	0.000057	-1.17	0.27	-2.23	0.00025

Rnr4 (d)	+2.41	0.0023	-1.06	0.74	+1.30	0.015

Ald6	-1.84	0.00056	-2.21	0.021	-1.81	0.00014

Bgl2	-1.85	0.00068	-1.54	0.17	+7.02	0.000022

Bmh1 (b)	-1.43	0.0020	-1.53	0.0071	-1.07	0.059

Bmh2	-1.90	0.0020	-2.08	0.021	-1.06	0.069

Hsp60	-1.72	0.00017	-1.56	0.091	+1.31	0.0079

Pdc1	-1.69	0.00022	-2.27	0.013	-2.64	0.000099

Rpc40	-1.53	0.0016	+2.31	0.013	+3.98	0.000015

Rpp0	-1.70	0.00056	-2.15	0.028	-1.61	0.00037

Ssb1	-1.62	0.0084	-1.41	0.12	-1.27	0.00027

Ssb2	-1.83	0.0092	+2.01	0.018	+1.45	0.0090

Tma19	-1.93	0.0027	-3.00	0.036	-1.02	0.14

Ura7	-1.85	0.0027	+6.01	0.059	+3.73	0.000022

Multiple protein isoforms are indicated with a, b, c and d in parentheses. DF, differential factor, is fold change in abundance in the chromatin fraction on MMS treatment, where +DF indicates an increase and -DF a decrease. EF is the chromatin enrichment factor relative to the whole cell extract in either treated (MMS+) or control (MMS-) samples. See Additional File 1, Table S2 for MS/MS identification data.

^a ^*p*-values are calculated using DeCyder 6.0 with FDR correction, from four biological replicates.

Along with the previously well-characterized proteins above, several additional DNA damage-associated proteins were identified as differentially expressed on MMS treatment including Bmh1, Pst2 Vma2, and Vma4 (see Table 2). Bmh1 is a 14-3-3 protein family member, which has been shown to directly modulate Rad53 activity [33]. Pst2, a predicted oxidative response protein, has also been implicated in DNA damage responses [29]. Vacuolar-type H+ ATPase subunits Vma2 and Vma4 have been shown to play a role in DNA damage responses following treatment with MMS and cisplatin [34]. In addition, several other proteins were identified that have not been well characterized in terms of their potential role following DNA damage, including Acf2, Arp3, Hsp31, Lsp1, Ste4, Ycp4, and Yrb1. Several proteins with low chromatin association (low EF values) and showing a differential response to MMS treatment were also identified (Table, 2, Additional File 1, Table S2). While these proteins are not chromatin associated per se, some (e.g. metabolic enzymes Ald6 and Pdc1) are consistent with a stress response in which yeast cells have a lowered metabolic activity and concomitant reduced growth competency. This observation is consistent with the model of suppressed protein synthesis upon DNA damage checkpoint execution or cellular stress [35]. It is also possible that for some of these factors the effect of MMS may not have been due to DNA damage, since this alkylating agent can also act directly on proteins [36,37]. A number of key DNA damage response factors including the kinases Mec1, Tel1, Rad53 and Chk1, and members of the 9-1-1 complex (Rad17, Mec3, Ddc1) (reviewed in [19]) were not among the proteins that we identified in this screen. However, this is not surprising as we characterized only a subset of proteins that were chromatin- and/or MMS-enriched in our samples.

### Changes in chromatin association and localization due to MMS treatment

**Figure 3:**
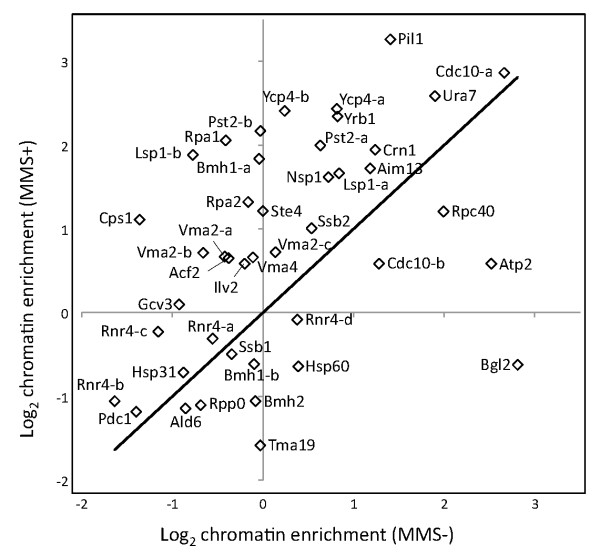
**Chromatin Enrichment Factors (EF) in the presence and absence of MMS**. Enrichment factors were calculated from the ratio of protein abundance in the chromatin fraction versus whole cell extract. A general increase in chromatin association is seen with MMS treatment, along with changes specific protein to given protein isoforms including Rnr4, Vma2, Pst2, Cdc10, Ycp4, Bmh1 and Lsp1.

The MMS DIGE experiment provided a direct measure of changes in protein abundance within the chromatin enriched fraction. This can represent a change in expression of the protein of interest, a change in the degree of chromatin association (including direct binding to DNA, interaction with DNA binding proteins, or simple inclusion in the chromatin pellet), or some combination of these factors. Here, the calculated EF ratios for MMS treated and control samples can be compared and changes in EF values can provide an estimate of changes in the degree of chromatin association (i.e. localization). The EF ratios for the control (MMS-) and treated (MMS+) samples are compared in Figure 3. The majority of proteins increased their degree of chromatin association in response to MMS treatment. Interestingly, different forms of the same protein often exhibited different changes in expression and chromatin association, including Rnr4, Vma2, Pst2, Lsp1, Ycp4, Cdc10 and Bmh1 (Table 2, Additional File 1, Table S2). For example, four isoforms of Rnr4 were detected, all of which increased in chromatin abundance in response to MMS treatment [Additional File 1, Figure S2]. Rnr4 was previously reported to undergo increased translocation to the cytoplasm under genotoxic stress [32]. Consistent with this, we find that the isoform with the highest chromatin association, Rnr4-d, showed a decrease in the proportion of Rnr4-d associated with chromatin on MMS treatment as reflected by the decrease in enrichment factor from +1.30 to -1.06. However, the total amount of all forms of Rnr4 binding chromatin increased, as all forms had positive DF values. The most abundant isoform, Rnr4-b [Additional File 1, Figure S2], had minimal association with chromatin with or without MMS treatment (Table 2). The observed values of DF and EF indicate a complex response, with some isoforms increasing and others decreasing relative degree of chromatin association (EF), while the total amount of cellular Rnr4 apparently increasing on MMS exposure.

All Vma2 isoforms demonstrated greater chromatin association (EF) as a consequence of MMS treatment, but isoforms Vma2-a and b, showed a more dramatic increase than Vma2-c. Comparing the DF and EF values in Table 2, the change in chromatin abundance can be largely attributed to an increase in chromatin association for Vma2 as opposed to increased cellular protein levels. Similarly, Rpa1 demonstrated a pronounced increase in chromatin association on MMS exposure, suggesting that the observed increase in chromatin abundance (DF) can be largely attributed to a change in cellular localization (Table 2). Conversely, Cdc10-b exhibited a small net increase in abundance in the chromatin fraction on MMS treatment (DF +1.38) but a decrease in EF from +2.43 to +1.50. This is consistent with an increase in cellular expression of Cdc10-b, but a smaller proportion of Cdc10-b associating with chromatin.

It has previously been observed that genes that are induced by DNA damaging agents are not those that are identified as protecting cells against DNA damage [38]. However, as proteins can respond more rapidly than genes through post-translational modifications or changes in localization, there may be a closer relationship between increased chromatin association and DNA-protective proteins. In contrast to gene expression data [38], we find that almost half of the proteins identified (10 of 22) were previously identified as responding to genotoxic agents in high-throughput screening studies. Specifically, *acf2*, *aim13*, *gcv3*, and *ycp4 *knockout strains were identified as having significant fitness defects (p < 0.05) on MMS exposure, with *aim13*, *bmh1*, *cdc10*, *cps1*, *gcv3*, *pil1*, *pst2*, *rnr4 *knockout strains having fitness defects on exposure to hydroxyurea [39].

### Evaluation of sensitivity to genotoxic agents for mutant yeast strains

To further investigate MMS induced proteins identified via the DIGE analysis, yeast strains with mutations corresponding to the genes encoding several of these proteins were evaluated in growth assays in the presence of the genotoxic agents MMS or HU [40,41]. Haploid cells either containing gene knockouts (*pst2*, *bmh1*, *hsp31*, *acf2*, *ste4*, *rnr4*) or, in the case of essential genes, lowered mRNA expression due to reduced mRNA stability, (DAmP strains, Open Biosystems) (*rpa1*, *rpa2*, *yrb1*, *arp3*) were employed. We used an isogenic wild-type strain as a negative control and a *rad53-11 *strain [42] with a mutant allele in the checkpoint kinase Rad53 as a positive control for sensitivity to genotoxic agents.

**Figure 4:**
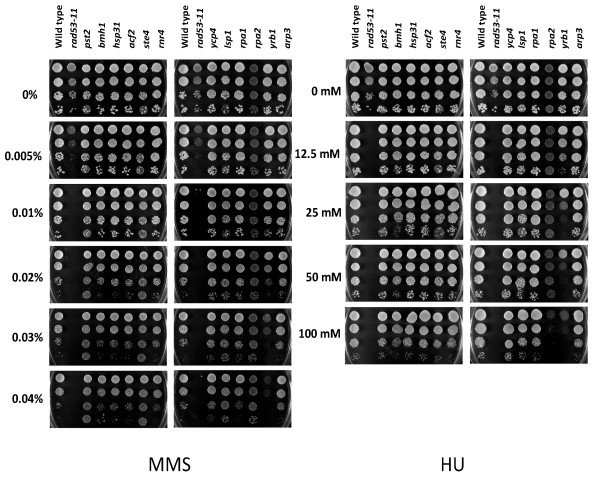
**Spotting growth assay for genotoxic sensitivity**. The yeast cells were either knockout (*ycp4, lsp1, pst2, bmh1, hsp31, acf2, ste4*, and *rnr4*) or DAmP strains (*rpa1*, *rpa2*, *yrb1 *and *arp3*). The assay was performed on YPD plates containing indicated concentrations of MMS or HU. Cells were 10-fold serially diluted and incubated at 30°C for 2 days. An isogenic wild-type strain BY4741 was used as a negative control and *rad53-11 *mutant strain as a positive control.

We were primarily interested in proteins increasing in chromatin abundance, however haploid yeast knockout strains corresponding to a number of proteins decreasing in abundance were also investigated [Additional File 1, Figure S3]. None of these strains showed either enhanced or reduced susceptibility to MMS or HU relative to the isogenic wild-type strain. Among the strains corresponding to proteins with increased chromatin abundance (Figure 4), *acf2*, *arp3*, *hsp31*, and *ycp4 *mutants did not show apparent changes in sensitivity relative to the wild-type strain. Interestingly, *pst2*, *ste4*, and *lsp1 *mutants actually exhibited increased resistance to MMS, indicating a link to the DNA damage response, possibly through interrelated pathways such as MAP kinase signaling, eisosome trafficking and oxidative stress response. *rpa1*, *rpa2 *and *rnr4 *mutants have previously been shown to be sensitive to MMS or HU, in agreement with their DNA damage checkpoint regulation [29-32]. Here however, no significant response was seen for *rpa1 *or *rpa2 *DAmP strains to MMS treatment and *rpa1 *to HU treatment. Subsequent western blot analysis of Rpa1 levels revealed that it was not reduced in the *rpa1 *DAmP strain relative to the isogenic wild-type (results not shown), accounting for the lack of sensitivity observed. Given that the *rpa2 *DAmP strain was sensitive to HU, its Rpa2 level presumably was reduced compared to wild-type, however it may not have been sufficiently diminished to render cells more vulnerable to the effects of MMS. The *rnr4 *knockout strain similarly did not show increased genotoxic sensitivity. It is possible that other *RNR *genes may compensate for *rnr4 *DAmP cells showed pronounced sensitivity to MMS (at more than 0.02%) and HU (at more than 25 mM). The Yrb1 protein in budding yeast has not been well characterized to date, but has been proposed to be a Ran-GTPase binding protein involved in nucleocytoplasmic transport [43].

## Conclusions

We have combined the differential proteomics technique of DIGE with a chromatin fractionation and enrichment strategy, and applied it to investigate the response to genotoxic agents in budding yeast cells. Our approach facilitated the selective screening of important chromatin-associated proteins that can otherwise be difficult to observe by typical proteomics approaches, and was successful in identifying functionally relevant target proteins. Moreover, the method was effective for the differential analysis of yeast cells following chemical treatment, as demonstrated by the MMS exposure experiment. While the fractionation method used was effective at enrichment of chromatin binding factors, a number of the observed proteins were likely mitochondrial, suggesting that more specific fractionation methods could be applied. One possible approach would be to first isolate nuclei prior to chromatin enrichment. Overall, the described method was successful in permitting the differential analysis of chromatin binding proteins using a gel-based proteomics technique, largely overcoming the technical limitations for analyzing lower-abundance chromatin proteins.

While the methodology was effective at identifying known and potentially novel proteins involved in DNA damage response, the technique does not provide comprehensive coverage. Future refinements to the methodology may be able to increase the number of factors identified in similar studies. The gel methods could be expanded to increase the pH range over which proteins can be separated effectively, more sensitive mass spectrometers may be used to increase the success rate of protein identification, a greater degree of replication and experimental precision may be utilized to detect proteins undergoing small changes in abundance and/or localization.

Characterization of differentially expressed proteins based on DF analysis was extended using an analysis of the chromatin enrichment factors (EF), providing a quantitative estimate of protein localization not typically available within proteomics studies. The method was also informative in addressing changes in protein localization, as demonstrated in the change of enrichment factor depending on treatment. Chromatin fractionation was able to consistently reveal a large population of chromatin-associated proteins using a relatively straightforward sampling procedure, in which intact complexes are maintained, as indicated by the co-detection of functionally related chromatin proteins (i.e. Tables 1 and 2). A technical strength of DIGE itself, compared to MS-based methods, is that it is able to distinguish differences in response to compounds such as MMS for different protein isoforms or post-translational variants, as revealed in the Rnr4 isoforms in this study. In contrast, quantitative MS-based methods largely rely on digested peptides [44], making it more challenging to distinguish variable forms, as the peptides on which the change is located need to be correctly identified, quantified, and compared with peptides representing other forms of the protein.

With respect to the budding yeast DNA damage response, this study was in broad agreement with previous high-throughput studies on this response, using a variety of approaches such as microarray analysis [45], phenotyping of deletion strains [46] and quantitative phosphoproteomics [5]. The microarray study showed the over-expression of the RNR complex (which is composed of four subunits Rnr1, Rnr2, Rnr3 and Rnr4) as the most significantly changed along with other key proteins such as Din7, Dun1, Rad54 and Rad51. The phosphoproteome study screened the possible phosphorylation-mediated targets of Mec1/Tel1 and Rad53 kinases [5], and identified proteins involved in DNA replication, cytokinesis, transcription, mitosis, RNA export, stress response, transcription, and nuclear transport. Compared to the above studies, our approach focused on a subset of the budding yeast proteome that is highly associated with chromatin. In addition to the confirmation of known checkpoint-regulated factors (e.g. Rpa1, Rpa2, Rnr4), several new proteins related to DNA damage response pathways have been identified. One such factor is the Ran-GTPase binding protein Yrb1, a component of the nuclear import-export system [43], in which the ternary complex of Gsp1, Yrb1 and Rna1 controls the GTP/GDP balance across the nuclear membrane. We propose here that Yrb1 protein may represent a link between the nuclear transport system and DNA damage responses, as implied by a recent model for G1-S cell cycle arrest during checkpoint execution [47]. It will now be of interest to determine which proteins dependent on Yrb1-mediated nucleocytoplasmic trafficking act downstream of this factor in affording protection to genotoxic agents.

In conclusion, we present a simple fractionation and DIGE-based approach for chromatin proteomics, which can be broadly applied to investigate biological responses to chemical stress and other factors. This method was successfully applied to investigate changes that occur following exposure to the genotoxic agent MMS, confirming that it is effective in identifying novel proteins involved in cellular processes, such as the response to DNA damage.

## Methods

### Yeast strains

Wild-type haploid *Saccharomyces cerevisae *strain BY4733 (MATa, *his3Δ*200, *leu2Δ*0, *met15Δ*0, *trp1Δ*63, *ura3Δ*0), purchased from Open Biosystems (Thermo Fisher Scientific Inc.), was used for all DIGE experiments. For genotoxic sensitivity assays, wild-type haploid strain BY4741 (MATa, *his3Δ*1, *leu2Δ*0, *met15Δ*0, *ura3Δ*0), isogenic MATa haploid DAmP strains YAR007c (*rpa1*), YNL312w (*rpa2*), YDR002w (*yrb1*), YJR065c (*arp3*), and isogenic haploid knock-out strains YCR004c (*ycp4*), YPL004c (*lsp1*), YDR032c (*pst2*), YER177w (*bmh1*), YDR533c (*hsp31*), YLR144c (*acf2*), YOR212w (*ste4*), YGR180c (*rnr4*) were purchased from Open Biosystems. A *rad53*-11 strain (MATa, *ura3, leu2, trp1, his3, rad53-11::URA3*) was used as a control for genotoxic sensitivity [42].

#### Cell culture

For large-scale cultures, a single colony was used to inoculate 10 ml YPD medium (1% yeast extract, 2% peptone, 2% glucose) in a 50 ml Falcon tube, which was then incubated at 30°C overnight with shaking. 10-20 *μ*l of saturated seed culture was transferred to 300 ml YPD medium in a 2 L flask and incubated with shaking at 30°C until a cell density of 2-3 × 10^7^/ml was achieved. For MMS treatment experiments, the 300 ml sample was centrifuged, the cell pellet was resuspended in 600 ml fresh YPD medium, and then divided equally into two 2 L flasks, and then further cultured at 30°C for 2 hrs. MMS was added to 0.03% for one of the flasks, and both were cultured at 30°C for another 90 min. The final cell density was not more than 3 × 10^7^/ml.

#### Chromatin preparation

Chromatin fractionation was based on the method of Liang and Stillman [11] with minor modifications. Cell cultures (300 ml in 2L flask) at a density of ~3 × 10^7^/ml were harvested in six 50 ml Falcon tubes and centrifuged at 4200 rpm for 5 min, washed with 40 ml dH_2_O. Cell pellets were resuspended in 5 ml 0.1 M EDTA-KOH (pH 8.0), 10 mM DTT, and incubated in a water bath for 15 min at 30°C. Cells were then centrifuged, resuspended in 5 ml of YPD, 1.1 M sorbitol 0.5 mg/ml Zymolase 20T™ (Sekagaku, Japan), 0.2 mg/ml Lyticase™ (Sigma), and incubated in a shaking water bath at 30°C for 20-30 min. Spheroplasts were collected by centrifugation at 2000 rpm for 3 min and washed once with 20 ml YPD, 1.1 M sorbitol, and 0.5 mM PMSF. The pellet was resuspended in 1 ml wash buffer (5 mM Tris-HCl (pH 7.4), 20 mM KCl, 2 mM EDTA, 0.12 mM spermidine, 0.05 mM spermine, 1 M sorbitol, 1% thiodiglycol, and Complete Mini™ EDTA-free protease inhibitor cocktail (1 tablet/10 ml) (Roche)) and transferred to two 1.5 ml centrifuge tubes on ice. Cells were centrifuged at 300 g for 1 min., washed twice with wash buffer, and centrifuged again. The pellet was then resuspended in 0.4 ml of lysis buffer (5 mM Tris-HCl (pH 7.4), 20 mM KCl, 2 mM EDTA, 0.12 mM spermidine, 0.05 mM spermine, 0.4 M sorbitol, 1% thiodiglycol, Complete Mini™ EDTA-free protease inhibitor cocktail (1 tablet/10 ml) (Roche)) and mixed with 0.5 ml of lysis buffer containing 2% (v/v) Triton X-100. For whole cell extracts (WCE), 200 μl of lysate from each sample was set aside and 2% SDS and 50 mM DTT added to maintain solubility. The remaining protein suspension was incubated on ice for a minimum of 10 min, followed by centrifugation at 16,000 g for 10 min to pellet chromatin, and the supernatant removed. The quality of the chromatin fractionation was verified by performing western blots for aliquots of the initial WCE, as well as chromatin and supernatant fractions, with antibodies for Orc2 and histone H2B which should both be chromatin-bound, as well as α-tubulin, which should be in the supernatant [11][Additional File 1, Figure S4].

#### Protein extraction

Chromatin pellets from the chromatin preparation were resuspended in two volumes of extraction buffer (50 mM Tris-HCl (pH 8.5), 2% (w/v) SDS, 50 mM DTT), and incubated in a boiling water bath for 10 min. Protein extracts were separated by centrifugation at 14,000 g for 10 min. The supernatants were collected then desalted using a 2-D Clean-Up kit (Amersham Biosciences). Protein pellets from the 2-D Clean-Up treatment were dissolved in IEF rehydration buffer (7 M urea, 2 M thiourea, 4% (w/v) CHAPS). Protein concentration was measured using the Bio-Rad protein assay (Bio-Rad). Protein yield of the chromatin fraction was calculated based on the amount in the chromatin preparation compared to the total amount in the WCE. A typical 300 ml culture at ~3 × 10^7^cells/ml yielded approximately 200 +/- 100 μg of protein in the chromatin fraction.

### Differential-in-gel-electrophoresis (DIGE)

DIGE was performed based on recommended protocols of the manufacturer (GE Healthcare) using minimal labeling CyDye™ DIGE Fluors of Cy2, Cy3 and Cy5. For the CyDye™ labeling reaction, 40 μg of protein sample in 50 μl of rehydration buffer containing 25 mM Tris-HCl (pH 8.5) was used for each dye. 1 μl CyDye™ solution (200 pmol/μl in 100% dimethylformamide) was added to samples on ice. The reaction was incubated for 40 min on ice, after which 1 μl of 10 mM lysine was added to stop the reaction. After incubation for 10 min, three sets of 50 μl samples (labeled with Cy2, Cy3 and Cy5) were combined and mixed with 45 μl of 1 M DTT, 4.5 μl of 1% (v/v) IEF buffer 4-7, 1 μl of 1% (w/v) bromophenol blue (BPB) and 250 μl of the rehydration buffer.

Immobiline™ DryStrip gels (IPG pH4-7/24 cm) (GE Healthcare) were used for isoelectric focusing as the first dimensional separation. The strips were passively rehydrated with 450 μl of labeled protein sample in the rehydration buffer overnight at room temperature. Isoelectric focusing (IEF) was performed using an Ettan™ IPGphor II system (GE Healthcare) with oil immersion and paper wicks at electrode contacts. The voltage profile used for IEF was as follows: hold at 500 V for 1 hr, gradient to 1,000 V for 3 hrs, gradient to 3,000 V for 3 hrs, hold at 3,000 V for 2 hrs, gradient to 8,000 V for 3 hrs, at 8,000 V for 10.5 hrs, and step to a final voltage of 500 V.

After the 1^st ^dimension separation, IEF strips were incubated in equilibration buffer (6 M urea, 2% (w/v) SDS, 50 mM Tris-HCl (pH 8.8), 30% (v/v) glycerol, 0.002% (w/v) BPB) containing DTT (10 mg/ml) for 20 min and then a further 20 min with the same buffer containing iodoacetamide (25 mg/ml). The strips were loaded onto 10% Tris-glycine SDS-polyacrylamide gels and run at 15 W per gel by using an Ettan™ DALTsix electrophoresis unit (GE Healthcare). Scanning of the DIGE gels was done using Typhoon 9400™ Variable Mode Imager (GE Healthcare).

We employed the three-dye system with four biologically independent replicates using an independent Cy2 dye channel as internal standard for each gel. The internal standard was composed of an equal mixture of control and test samples. The control and test samples used either Cy3 or Cy5 with dye swapping. Gel image analysis was performed using DeCyder™ 2-D differential analysis software version 6.0 (GE Healthcare), with the peak detection threshold set to an expected value of 2500 spots. Protein spots were quantified using peak volumes calculated by the DeCyder™ software. Each gel was normalized based on the independent Cy2 channel using the differential in-gel analysis (DIA) module. Biological variation analysis (BVA) was done for four replicates, including 4 internal standards, 4 controls and 4 test samples. Statistical analysis of spots was performed by the Student's t-test with FDR (false discovery rate) correction as previously described [48,49]. Average spot ratios for treated to control samples were calculated based on spot volumes for each matched spot, along with *p*-values. For the chromatin enrichment analysis, we defined the average ratio normalized spot volumes for the chromatin fraction vs. WCE as the enrichment factor (EF). EF values were calculated by the DeCyder software as fold change (EF = chromatin abundance/WCE) when chromatin abundance exceeded WCE abundance for the target protein, and as a negative fold change (EF = -WCE/chromatin abundance) otherwise. For experiments comparing the MMS-treated vs. non-treated chromatin fraction, differential factors (DF) were similarly calculated, where DF = (MMS treated/control) when treated ≥ control, and as a negative fold change (DF = -control/MMS treated) otherwise. As with the chromatin abundance experiment, the average ratio of spot volumes of the two DIGE channels being compared is reported. Calculated enrichment factors (EF) provide a measure of chromatin association (protein localization) independent of total protein abundance, whereas the differential factors (DF) measure changes in abundance in the chromatin fraction, including both changes in total abundance in the cell and changes in protein localization. For graphical presentation, a log scale is used and values are presented as log_2 _(treated/control). To determine the magnitude of change that is likely to be detected, a post-hoc power analysis was conducted using the statistical analysis package R [50]. Standard deviations were calculated for all spots appearing on 10 or more gel image channels (i.e. 10 from 12 total on 4 gels), and used to estimate the expected detectable fold change with a power of 0.80 (β = 0.20).

### Preparative 2D-PAGE

For preparative 2D-PAGE, 0.7 to 1.0 mg protein was separated on large-format gels using a 24 cm IPG 4-7 strip for 1^st ^dimension separation and an SDS-PAGE gel for the second dimension as described above. Preparative gels were visualized by the colloidal Coomassie-staining method [51] and scanned using a Typhoon 9400™ Variable Mode Imager (GE Healthcare). Spots of interest were matched between DIGE images and the preparative gel, and spots manually excised for protein identification. Spots were prioritized for identification using an FDR corrected *p*-value cut-off of 0.05 and a change in expression of 1.4 or greater.

### Mass spectrometry

Protein spots excised from the preparative gel were cut into approximately 1 mm^3 ^pieces, then reduced and alkylated by treatment with 10 mM DTT and 55 mM iodoacetamide in 50 mM ammonium bicarbonate buffer [52]. Gel pieces were washed with 50 mM ammonium bicarbonate buffer and dehydrated in a SpeedVac^® ^concentrator (Savant) for 1 hr, soaked with 3-10 μl of 20 ng/μl trypsin solution (sequencing grade modified trypsin, Promega) in 50 mM acetic acid on ice for 20 min, then washed again with buffer. Protein digestion was performed in the same buffer (50 μl) overnight at 35°C. Reaction supernatant was recovered and gel pieces were further extracted by 2× sonication in 50 μl 50% acetonitrile/1% trifluroacetic acid, and then dried using a SpeedVac^® ^concentrator (Savant). Mass spectrometry for higher abundance spots was performed using a Waters micromass quadrupole time of flight (Q-TOF) Ultima mass spectrometer with a nanospray ESI injection at mass spectrometry facility in University of Waterloo. Samples analyzed using the Q-Tof were desalted prior to analysis using C_18 _ZipTip^® ^pipette tips (Millipore) and eluted using 50% acetonitrile in water with 0.2% formic acid. For lower abundance spots, trypsin-digested peptides were analyzed (without ZipTip desalting) using an Applied Biosystems Q-Trap mass spectrometry system at the Proteomics Core Facility of Dalhousie University (Halifax, Nova Scotia).

#### Protein identification

Protein identification was performed using Peaks Studio (version 2.4, Bioinformatics Solutions, Waterloo), which combines auto *de novo *sequencing and homology-based database searching, with the non-redundant MSDB database (Dr. D.N Perkins, Imperial College London, Release 20063108, 3239079 sequences). Mass error tolerances of parental and fragment ions were set at 0.1 for Q-TOF spectra and 0.3 or 0.4 for Q-Trap spectra, with 0.3 used if a more restrictive search was required. Confidence of protein identifications were based on the Peaks database search score (%) according to the algorithm of Ma et al. (2005) [53]. Protein identifications were accepted if the homology search score was higher than 80% (i.e. extremely high confidence) and identified a single yeast protein. For Peaks scores less than 80%, protein identity was additionally confirmed using the web-based Mascot search engine (version 4) in the MS/MS ion search module (Matrix Science, http://www.matrixscience.com) with MSDB by restricted to *Saccharomyces cerevisiae *(10742 sequences) as the target organism. Protein matches were retained if the Mascot search had a significance threshold of p < 0.05, with default mass error tolerances of 1.2 and 0.6 Da for parental and fragment ions, respectively. Finally, the false discovery rate (threshold set to 0.01) was confirmed by performing the Mascot decoy database search. For both the Peaks and Mascot database searches, trypsin was set as the digestive protease allowing one missed cleavage, and carbamidomethylation of cysteine and oxidation of methionine were set as the fixed and variable modifications, respectively. For counting number of unique peptides matching to hit proteins, only peptide ions that are doubly or triply charged were included. Peptide sequence coverage (%) was obtained based on the matching peptide sequences from Peaks.

#### Genotoxic sensitivity assays

To identify potential genotoxic effects of targeted proteins, a spotting growth assay was performed to assess MMS or hydroxyurea (HU) resistance [40,41] in gene knockout cell lines or cells with lowered mRNA expression. Haploid knockout and DAmP cell lines in a BY4741 background were purchased from Open Biosystems (Thermo Fisher Scientific Inc.). BY4741 wild type and DNA damage checkpoint compromised rad53-11 [42] strains were used as controls. Cultures of cells were grown to saturation (~2 × 10^8 ^cells/ml) and serial 10-fold dilutions, ranging from 10^7 ^cells/ml to 10^4 ^cells/ml, were prepared for each strain. 5 μl of each dilution was spotted onto a series of YPD plates with varying concentrations of MMS (up to 0.04%) or HU (up to 100 mM). The plates were incubated at 30°C for 2 days.

## Competing interests

The authors declare that they have no competing interests.

## Authors' contributions

DRK conducted the proteomics analysis and yeast genetics experiments and drafted the manuscript. RDG assisted with proteomics analysis and yeast genetics experiments. BPI, BPD, and BJM conceived the study, assisted with experimental design, and contributed to preparation of the manuscript. All authors have read and approved the final manuscript.

## Supplementary Material

Additional file 1 **Supplementary figures 1-4, Supplementary Tables 1 and 2**. **Figure S1**. DIGE gel image comparing chromatin fraction and whole cell extract. **Figure S2**. DIGE gel image comparing MMS treated and control chromatin fractions. **Figure S3**. Additional spotting growth assay for genotoxic sensitivity. **Figure S4**. Western blot analysis of chromatin fractionation samples. **Table S1**. Mass spectrometry data for proteins identified in chromatin enriched sample. **Table S2**. Mass spectrometric identification of MMS-induced differentially expressed proteins. Click here for file
