# Supplementary material for: Differential chromatin proteomics of the MMS-induced DNA damage response in yeast
Source: Proteome Sci. 2011 Oct 4;9:62. doi: 10.1186/1477-5956-9-62 (PMC3212819; doi:10.1186/1477-5956-9-62)
Supplement: Additional file 1 — Supplementary figures 1-4, Supplementary Tables 1 and 2. Figure S1. DIGE gel image comparing chromatin fraction and whole cell extract. Figure S2. DIGE gel image comparing MMS treated and control chromatin fractions. Figure S3. Additional spotting growth assay for genotoxic sensitivity. Figure S4. Western blot analysis of chromatin fractionation samples. Table S1. Mass spectrometry data for proteins identified in chromatin enriched sample. Table S2. Mass spectrometric identification of MMS-induced differentially expressed proteins. [file 1477-5956-9-62-S1.PDF]

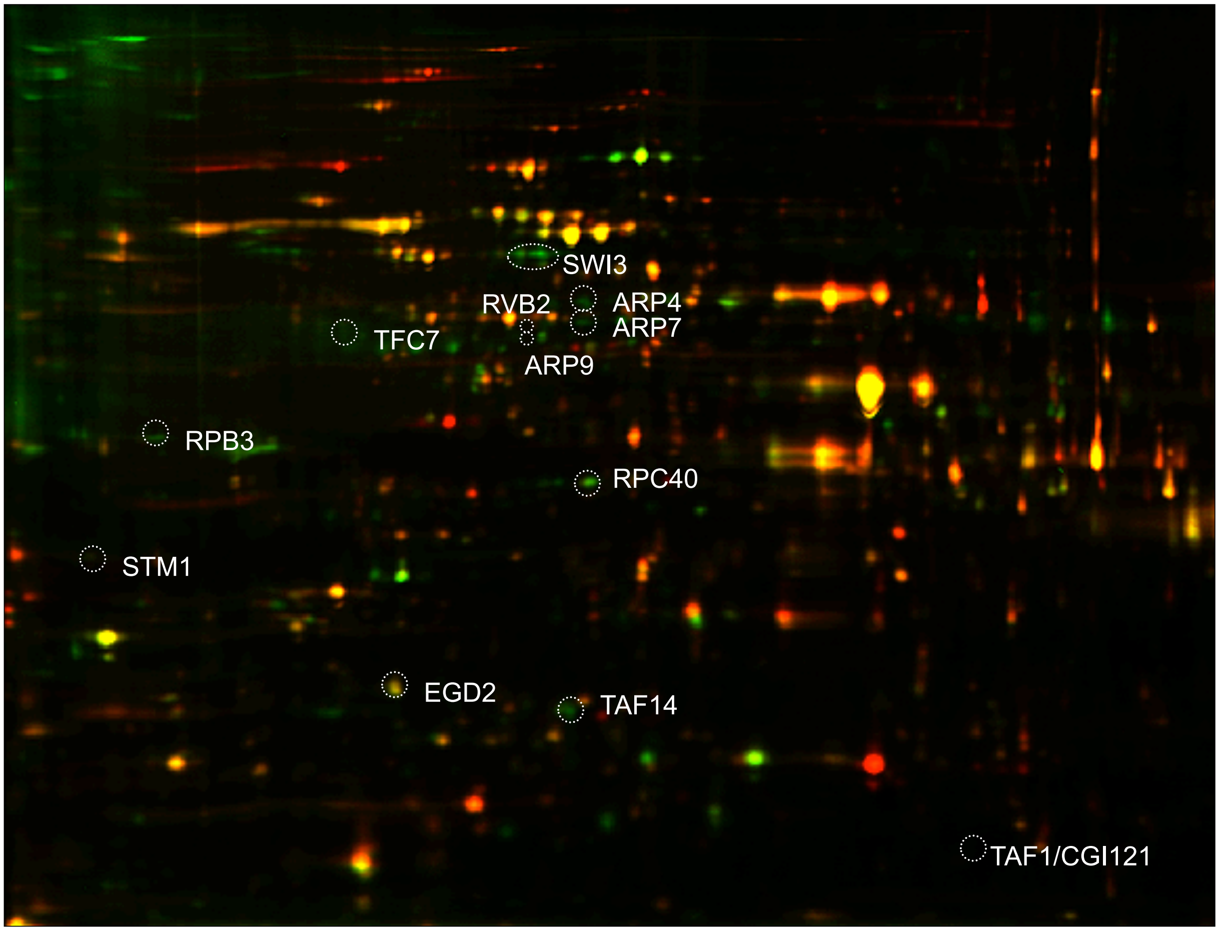

Suppl. Figure 1. DIGE gel image comparing a chromatin fraction (green, Cy3) and whole cell extract (red, Cy5). The Cy2 channel used for the internal control is not shown. Four replicates of biologically independent samples were tested in four different gels with two by two dye swapping. The chromatin-enriched spots appear predominantly green in this representative image. Selected identified proteins with known chromatin-association are indicated.

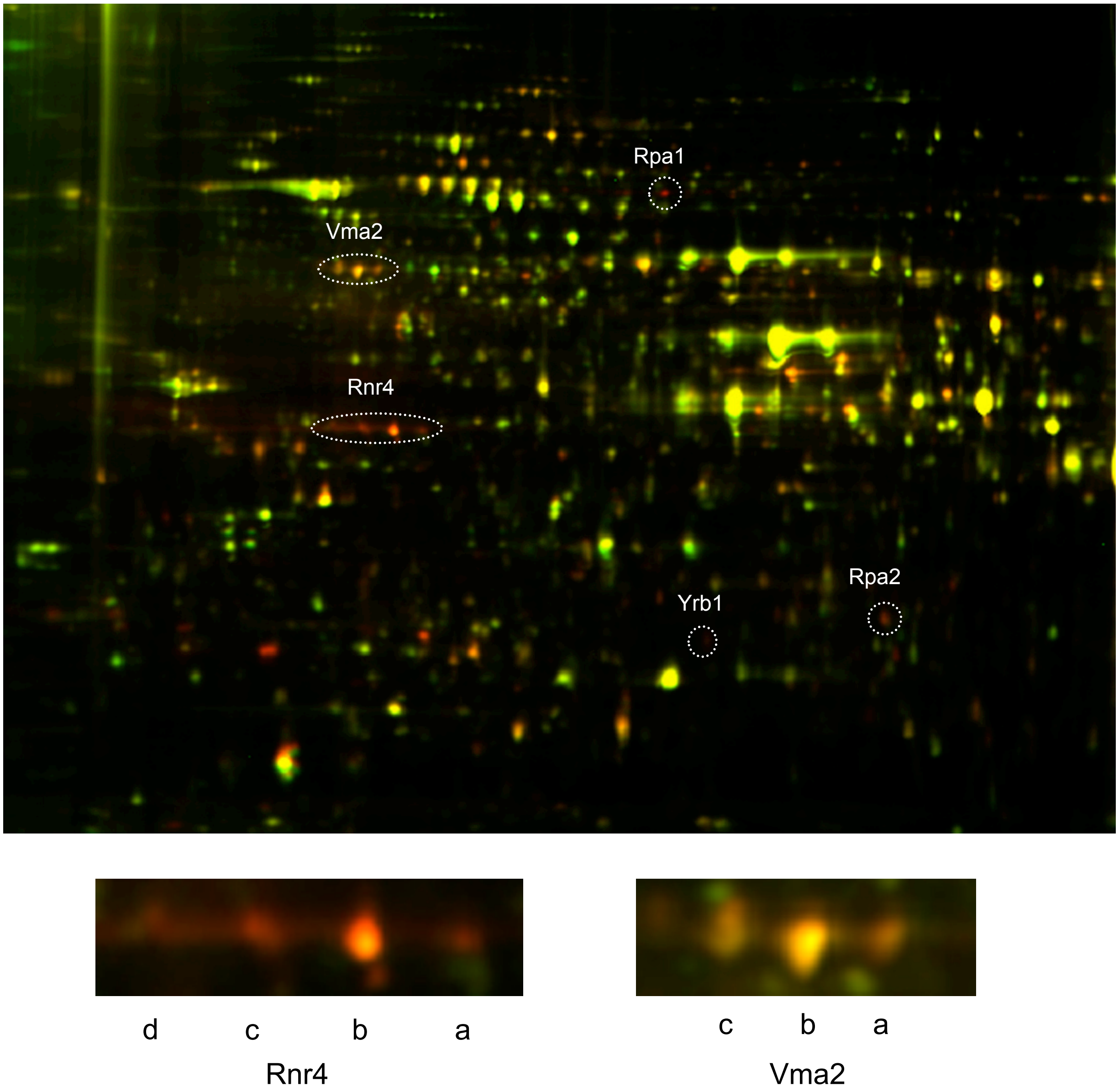

Suppl. Figure 2. DIGE gel image comparing MMS treated and control chromatin fractions. A gel image from one of four replicates is shown for MMS-treated (red, Cy5) and untreated (green, Cy3) chromatin fractions. The Cy2 channel used for internal control is not shown. Five representative proteins (Rnr4, Rpa1, Rpa2, Vma2, Yrb1) are indicated along with an expansion of the region showing multiple identified isoforms for Rnr4 and Vma2.

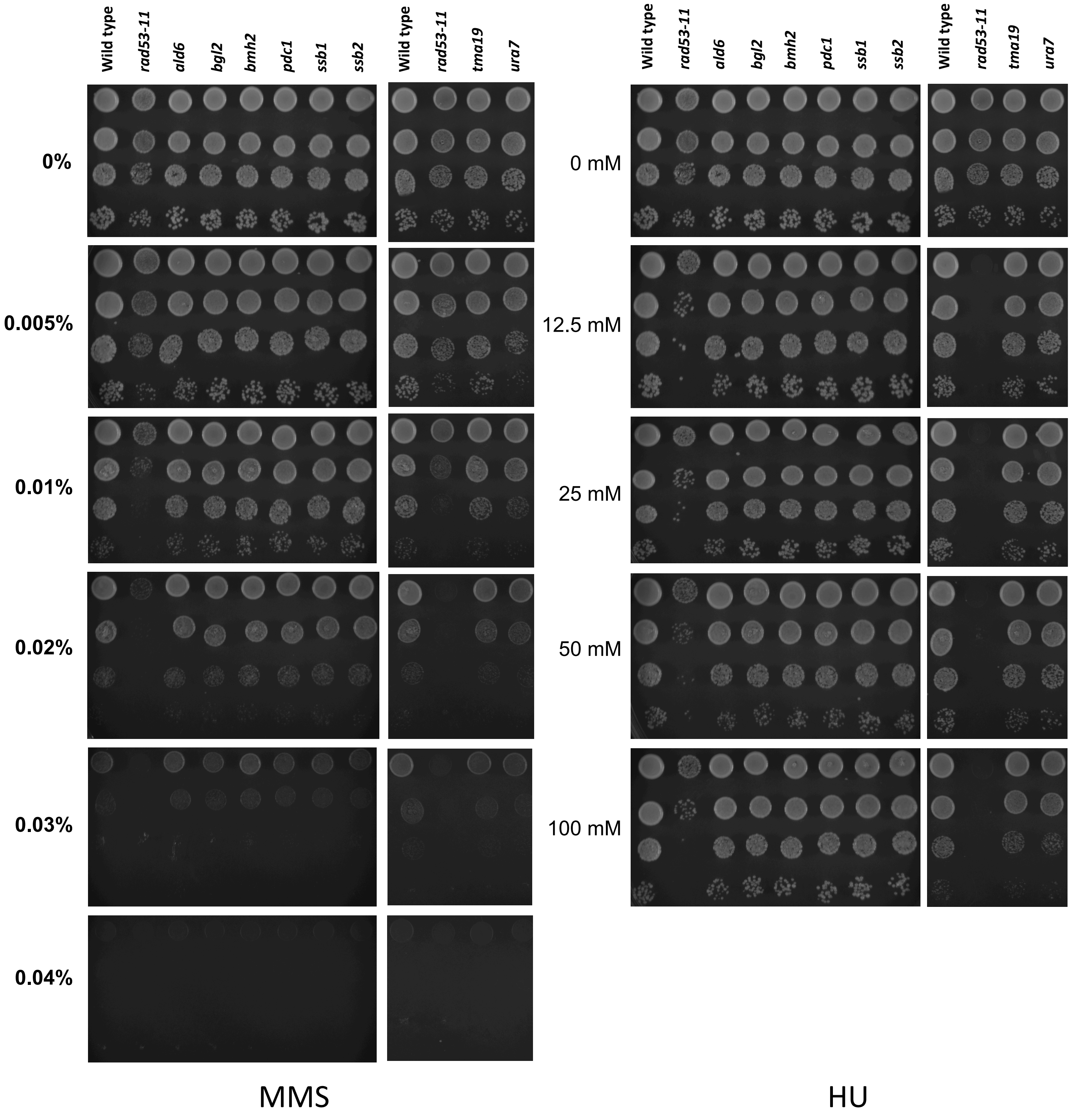

Suppl. Figure 3. Additional spotting growth assay for genotoxic sensitivity. Haploid yeast knockout strains corresponding to proteins that showed a reduction in chromatin association following MMS exposure were obtained from Open Biosystems. Wild-type, *rad53-11*, *ald6*, *bgl2*, *bmh2*, *pdc1*, *ssb1*, *ssb2*, *tma19* and *ura7* strains were analyzed as described in Figure 4.

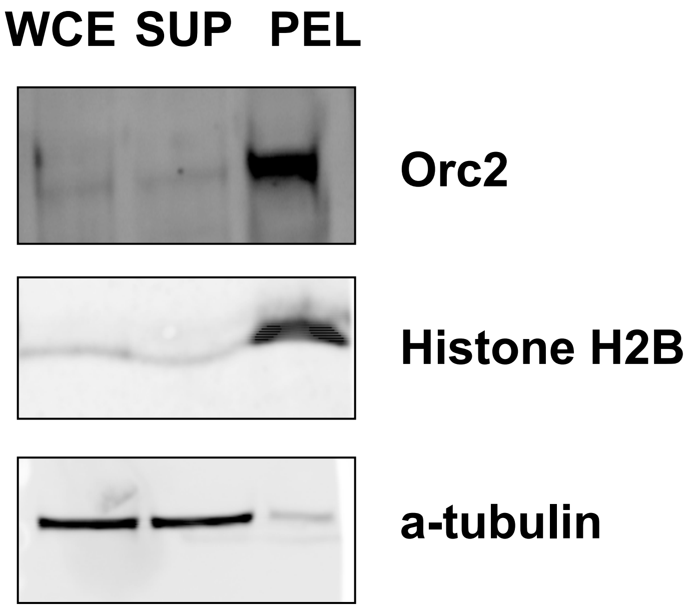

Suppl. Figure 4. Western blot analysis of chromatin fractionation samples. Initial whole cell extract (WCE), as well as supernatant (SUP) and chromatin (PEL) fractions were subjected to SDS-PAGE and transferred to a nitrocellulose membrane. Detection was carried out with rabbit anti-Orc2 (1:1000 dilution, Duncker et al., 2002), mouse anti-α-tubulin (1:500 dilution, Sherwin and Gull, 1989), and rabbit anti- histone H2B (1:1000 dilution, Active Motif), using 1:3000 dilutions of either Alexa Fluor 647 goat anti-rabbit IgG or Alexa Fluor 488 goat anti-mouse IgG secondary antibodies. PEL fractions are concentrated tenfold relative to WCE and SUP. In each case, equal volumes of WCE and SUP fractions were loaded, with double (histone H2B, α-tubulin detection) or triple (Orc2 detection) the volume of the PEL fraction loaded. This resulted in approximately equal amounts of protein being analyzed for each sample.

Supplementary references:

Duncker, B.P., Shimada, K., Tsai-Plugfelder, M., Pasero, P. and Gasser, S.M. (2002) An N-terminal domain of Dbf4p mediates interaction with both origin recognition complex (ORC) and Rad53p and can deregulate late origin firing. Proc. Natl. Acad. Sci. USA 99, 16087-16092

Sherwin, T. and Gull, K. (1989) Visualisation of detyrosination along single microtubules reveals a novel mechanism of assembly during cytoskeletal duplication in trypanosomes. Cell 57, 211-221

Suppl. Table 1. Mass spectrometry data for proteins identified in chromatin enriched sample.

Protein name	Systematic name[a]	Peaks score / sequence coverage (%) (Mascot score)[b]	Peptides matched	Mw (kDa)	EF	p-value[c]
Arp3	YJR065c	98.90 / 14.03	6	49.49	+1.74	0.0043
Arp4	YJL081c	99.97 / 31.49	17	54.78	+4.20	0.0010
Arp7	YPR034w	94.41 / 20.13	9	53.70	+2.53	0.0011
Arp9	YMR033w	46.06 / 5.35 (63)	2	53.02	+3.35	0.00035
Asc1	YMR116c	81.04 / 23.20	6	34.63	+2.88	0.0046
Atp2	YJR121w	99.99 / 38.36	15	54.74	+1.43	0.00025
Cdc10	YCR022c	97.10 / 17.38	5	40.00	+3.09	0.0045
Cgi121	YML036w	39.73 / 13.81 (73)	3	20.63	+3.37	0.00030
Crn1	YLR429w	97.65 / 8.29	5	72.49	+4.06	0.000025
Cys3	YAL012w	98.23 / 17.77	6	42.50	+1.65	0.00076
Egd2	YHR193c	54.70 / 15.50 (56)	2	18.68	+1.55	0.0016
End3	YNL084c	63.53 / 10.03 (109)	3	40.28	+10.4	0.00012
Gdi1	YER136w	96.87 / 22.20	9	51.16	+1.58	0.00019
Ilv2	YMR108w	93.25 / 9.17	5	74.87	+13.4	0.000031
Ilv5	YLR355c	96.35 / 25.37	7	44.30	+1.95	0.00050
Lat1	YNL071w	92.00 / 14.11	6	51.76	+2.42	0.00097
Lsp1	YPL004c	98.28 / 21.99	6	38.03	+5.54	0.00043
Pdb1	YBR221c	97.14 / 20.22	6	40.02	+3.93	0.000036
Pil1 (a)	YGR086c	87.21 / 31.27	8	38.31	+1.65	0.0029
Pil1 (b)	YGR086c	94.80 / 14.45	5	38.31	+2.31	0.00061
Pst2	YDR032c	74.95 / 12.12 (97)	2	20.93	+3.37	0.000062
Qcr2	YPR191w	98.72 / 26.90	8	40.44	+1.73	0.00036
Raf1	R0030w	98.33 / 32.04	6	21.26	+3.37	0.00030
Rpb3	YIL021w	90.73 / 20.44	5	35.26	+3.38	0.000022
Rpc40	YPR110c	97.49 / 26.27	8	37.65	+3.98	0.000015
Rpt1	YKL145w	84.18 / 19.06	9	51.93	+1.92	0.00091
Rvb2	YPL235w	29.20 / 9.10 (54)	5	51.56	+4.03	0.00055
Stm1	YLR150w	44.40 / 18.32 (220)	4	30.00	+2.21	0.000082
Swi3	YJL176c	96.50 / 14.20	4	63.11	+6.85	0.000065
Taf14	YPL129w	95.30 / 14.30	4	27.40	+4.32	0.000065
Tfc7	YOR110w	36.10 / 8.10 (78)	3	49.10	+3.89	0.000027
Tub2	YFL037w	70.61 / 6.64 (53)	2	50.58	+3.73	0.0010
Ume1	YPL139c	95.57 / 17.39	4	50.97	+2.22	0.00020
Ura7	YBL031c	67.53 / 3.45 (76)	2	64.65	+3.73	0.000022

[a] Saccharomyces Genome Database (SGD) identifier
[b] Protein identification score as described in Ma *et al*. (2005) [53]. Score and sequence coverage are calculated using Peaks Studio version 2.4. If the Peaks search score was below the threshold of 80, additional confirmation was made using the Mascot MS/MS ion search with a confidence cutoff of $p<0.05$ (Mascot scores shown in parentheses).
[c] *p*-values of DIGE experiments are obtained with Decyder FDR correction with four biological replicates.

Suppl. Table 2. Mass spectrometric identification of MMS-induced differentially expressed proteins.

Protein name	Description	Systematic name[a]	MS/MS score /sequence coverage (%)[b]	Peptides matched	Mw (kDa)
Acf2	Cytoskeleton assembly factor	YLR144c	99.71 / 20.41	15	88.00
Aim13	Genome stability protein	YFR011c	85.27 / 27.06	5	18.83
Arp3	Actin-related protein 3	YJR065c	99.93 / 51.45	17	49.49
Atp2	ATPase beta chain	YJR121w	95.64 / 38.16	41	54.74
Bmh1 (a)	14-3-3 homolog	YER177w	99.64 / 70.41	30	30.05
Cdc10 (a)	Septin ring protein, cytokinesis	YCR002c	99.99 / 61.80	24	36.98
Cdc10 (b)	Septin ring protein, cytokinesis	YCR002c	97.12 / 17.39	6	36.98
Cps1	Gly-X carboxypeptidase	YJL172w	98.72 / 43.58	72	64.54
Crn1	Coronin	YLR429w	92.81 / 26.27	13	72.49
Gcv3	FUN40, glycine cleavage	YAL044c	94.10 / 47.46	13	19.55
Hsp31	Cysteine-type endopeptidase	YDR533c	99.78 / 66.24	16	25.64
Ilv2	Acetolactate synthase	YMR108w	99.99 / 39.88	32	74.87
Lsp1 (a)	Primary component of eisosome	YPL004c	99.31 / 25.81	8	38.03
Lsp1 (b)	Primary component of eisosome	YPL004c	77.31 / 17.3 (34)	6	38.03
Nsp1	Nucleoskeletal like protein	YJL041w	99.99 / 28.68	24	86.50
Pil1	Component of eisosome	YGR086c	94.80 / 14.45	5	38.31
Pst2 (a)	Flavodoxin-like protein	YDR032c	90.38 / 33.84	5	20.93
Pst2 (b)	Flavodoxin-like protein	YDR032c	94.19 / 52.02	6	20.93
Rpa1	Replication factor A 1	YAR007c	100.00 / 40.10	22	70.29
Rpa2	Replication factor A 2	YNL312w	82.44 / 37.0	6	29.90
Rnr4 (a)	Ribonucleotide reductase	YGR180c	99.02 / 43.77	11	39.98
Rnr4 (b)	Ribonucleotide reductase	YGR180c	99.83 / 33.33	19	39.98
Rnr4 (c)	Ribonucleotide reductase	YGR180c	81.68 / 22.03	9	39.98
Rnr4 (d)	Ribonucleotide reductase	YGR180c	97.64 / 31.59	12	39.98
Ste4	GTP binding protein	YOR212w	71.56 / 14.18 (113)	4	46.53
Vma2 (a)	Subunit 2 of V-ATPase	YBR127c	99.40 / 24.76	10	57.70
Vma2 (b)	Subunit 2 of V-ATPase	YBR127c	99.82 / 28.82	13	57.70
Vma2 (c)	Subunit 2 of V-ATPase	YBR127c	99.35 / 24.76	13	57.70
Vma4	Subunit 4 of V-ATPase	YOR332w	99.82 / 27.90	9	26.44
Ycp4 (a)	Flavodoxin-like protein	YCR004c	99.57 / 43.81	7	26.32
Ycp4 (b)	Flavodoxin-like protein	YCR004c	87.20 / 17.41(169)	3	26.32
Yrb1	Ran GTPase binding protein	YDR002w	52.21 / 26.37 (38)	6	22.92
Ald6	Aldehyde dehydrogenase	YPL061w	99.79 / 25.75	10	54.52
Bgl2	Glucan endo-1,3-beta-glucosidase	YGR282c	99.49 / 18.61	9	33.50
Bmh1 (b)	14-3-3 protein 1	YER177W	99.59 / 39.70	9	30.05
Bmh2	14-3-3 protein 2	YDR088w	97.78 / 25.64	6	31.13
Hsp60	Mitochondrial chaperone	YLR259c	99.90 / 26.22	12	60.69
Pdc1	Pyruvate decarboxylase	YLR044c	99.88 / 25.22	13	61.44
Rpp0	Acidic ribosomal protein P0	YLR340w	98.83 / 24.68	7	33.68
Rpc40	Component of RNA polymerase	YPR110c	98.10 / 19.70	6	37.65
Ssb1	DnaK-type molecular chaperone	YDL229w	99.86 / 21/21	9	66.54
Ssb2	DnaK-type molecular chaperone	YNL209w	99.74 / 26.10	13	66.54
Tma19	Histamine-releasing factor homolog	YKL056c	67.11 / 23.35 (69)	3	18.71
Ura7	CTP synthase	YBL039c	67.53 / 3.45 (76)	2	64.65

[a] Saccharomyces Genome Database (SGD) identifier

[b] Protein identification score as described in Ma *et al*. (2005) [53]. Score and sequence coverage are calculated using Peaks Studio version 2.4. If the Peaks search score was below the threshold of 80, additional confirmation was made using the Mascot MS/MS ion search, and the identification retained for Mascot scores significant at $p<0.05$ (Mascot score shown in parentheses).
